# Supplementary material for: Quantifying Emission Factors and Setting Conditions of Use According to ECHA Chapter R.14 for a Spray Process Designed for Nanocoatings—A Case Study
Source: Nanomaterials (Basel). 2022 Feb 10;12(4):596. doi: 10.3390/nano12040596 (PMC8876979; doi:10.3390/nano12040596)
Supplement: Supplementary file 1 [file nanomaterials-12-00596-s001.zip › nanomaterials-1531087 - supplementary 1-proof done.pdf]

nanomaterials

Supplementary Material 1

Quantifying Emission Factors and Setting Conditions of Use According to ECHA Chapter R.14 for a Spray Process Designed for Nanocoatings—A Case Study

Antti Joonas Koivisto [1,2,3,*], Benedetta Del Secco [4], Sara Trabucco [4], Alessia Nicosia [4], Fabrizio Ravegnani [4], Marko Altin [5], Joan Cabellos [6], Irini Furxhi [7,8], Magda Blosi [9], Anna Costa [9], Jesús Lopez de Ipiña [10] and Franco Belosi [4]

1 Air Pollution Management APM, Mattilanmäki 38, 33610 Tampere, Finland
2 Institute for Atmospheric and Earth System Research (INAR), University of Helsinki, PL 64, FI-00014 Helsinki, Finland
3 ARCHE Consulting, Liefkensstraat 35D, B-9032 Wondelgem, Belgium
4 CNR-ISAC, Institute of Atmospheric Sciences and Climate, National Research Council of Italy, Via Gobetti, 101, 40129 Bologna, Italy; b.delsecco@isac.cnr.it (B.D.S.); s.trabucco@isac.cnr.it (S.T.); a.nicosia@isac.cnr.it (A.N.); f.ravegnani@isac.cnr.it (F.R.); f.belosi@isac.cnr.it (F.B.)
5 Witek srl, Via Siena 47, 50142 Firenze, Italy; marko.altin@witekgroup.com
6 Leitat Technological Center, c/de la Innovació 2, Terrassa, 08225 Barcelona, Spain; jcabellos@leitat.org
7 Transgero Limited, Cullinagh, Newcastle West, Co. Limerick, V42 V384 Limerick, Ireland; irini.furxhi@transgero.eu
8 Department of Accounting and Finance, Kemmy Business School, University of Limerick, V94 T9PX Limerick, Ireland
9 ISTEC-CNR, Institute of Science and Technology for Ceramics, CNR, National Research Council, Via Granarolo 64, 48018 Faenza, Italy; magda.blosi@istec.cnr.it (M.B), anna.costa@istec.cnr.it (A.C.)
10 Basque Research and Technology Alliance (BRTA), Consiglio Nazionale delle Ricerche, Parque Tecnológico de Alava, Leonardo Da Vinci 11, 01510 Miñano, Spain; jesus.lopezdeipina@tecnalia.com (J.L.d.I.)
* Correspondence: joonas.apm@gmail.com; Tel: +358-407-222-029

TEXT S1: Near-field volume

Version: TEAS 2019 (Version 1.0)

Date: 5/5/2021 11:22:04 AM

Prediction Model: Near Field Volume

Variable	Units	Variable Model
Side Length	m	~ Triangular (1.5, 1, 2)

Near Field Shape: Cube (square box)

STATISTICS

Simulation N: 50000

Non-parametric Statistics

Min: 1.009

Max: 7.982

Median: 3.373

Mode: 3.171

X0.05: 1.564

X0.95: 6.243

Normal Distribution Statistics

Mean: 3.563

SD: 1.414

X0.05: 1.237

X0.95: 5.889

Lognormal Distribution Statistics

GM: 3.282

GSD: 1.514

Mode: 2.763

X0.05: 1.659

X0.95: 6.492

GRAPHS

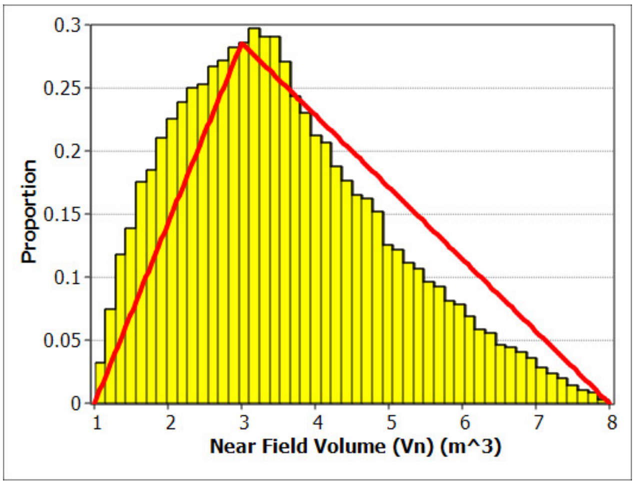

APPROXIMATE STATISTICAL MODEL

V_N ~ Triangular(3.0, 1.00, 8) m^3

NOTES

SOURCE:
-Nicas (2009): Chapter 6 - The Near Field/Far Field (Two Box) Model with a Constant Contaminant Emission Rate. in Mathematical Models for Estimating Occupational Exposure to Chemical, 2nd Edition. AIHA.

NOTES:

1. Box - the worker is assumed to be standing at the front.

2. Beta = 1/2 * (Free Surface Area) * S

3. Free Surface Area does not include the blocked surfaces.

TEXT S2: Near-field flowrate

Version: TEAS 2019 (Version 1.0)

Date: 5/5/2021 11:45:19 AM

Prediction Model: Near Field Flowrate (ß)

Variable	Units	Variable Model
Side Length	m	~ Triangular (1.5, 1, 2)
Air Velocity	m/min	~ Triangular (0.9, 0.45, 1.8)

Near Field Shape: Cube (square box)

Blocked Sides: Side

STATISTICS

Simulation N: 50000

Non-parametric Statistics

Min: 1.293

Max: 16.874

Median: 5.666

Mode: 4.876

X0.05: 2.858

X0.95: 10.383

Normal Distribution Statistics

Mean: 6.017

SD: 2.318

X0.05: 2.203

X0.95: 9.831

Lognormal Distribution Statistics

GM: 5.588

GSD: 1.477

Mode: 4.800

X0.05: 2.943

X0.95: 10.612

GRAPHS

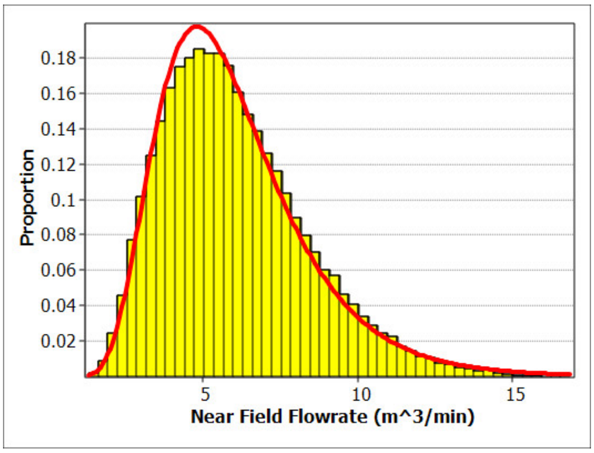

APPROXIMATE STATISTICAL MODEL

Beta ~ Lognormal(5.588, 1.477) m^3/min

NOTES

SOURCE:
-Nicas (2009): Chapter 6 - The Near Field/Far Field (Two Box) Model with a Constant Contaminant Emission Rate. in Mathematical Models for Estimating Occupational Exposure to Chemical, 2nd Edition. AIHA.

NOTES:
1. Box - the worker is assumed to be standing at the front.

2. Beta = 1/2 * (Free Surface Area) * S

3. Free Surface Area does not include the blocked surfaces.

TEXT S3: TiO_2-N Near-Field

Version: TEAS 2019 (Version 1.0)
Date: 6/24/2021 3:17:45 PM

SUBSTANCE INFORMATION

TiO_2-N particles dispersed in EtOH (1% w/w). TiO_2 content of aerosol particles at NF was 44% according to the ICP-MS analysis of collected particles.

EXPOSURE LIMITS

Limit value	Type	Source	Notes
REL: 0.3 mg/m3	TWA	NIOSH REL	Here concentrations are for total dust (44% is TiO_2 from the total dust)

TASK INFORMATION

TASK 1 - Break
Description:
Model: M201
Model Name: 2Box.CE.Gv
Comments:

Variable	Variable Model	Units
1: Frequency	~ ConstantInt (1, ,)	/shift
2: Duration	~ UniformInt (18, 19,)	min
3: G	~ Constant (0, ,)	mg/min
4: Q	~ Uniform (2.5, 10,)	ACH
5: V	~ Uniform (1200, 1470,)	m^3
6: Vn	~ Triangular (3, 1, 8)	m^3

7: ß	~ Lognormal (5.588, 1.477,)	m^3/min
8: tg	~ Constant (0, ,)	min
9: t1	~ Constant (0, ,)	min
10: t2	~ Constant (19, ,)	min

TASK 2 - T1

Description:

Model: M201

Model Name: 2Box.CE.Gv

Variable	Variable Model	Units
1: Frequency	~ ConstantInt (4, ,)	/shift
2: Duration	~ UniformInt (7, 9,)	min
3: G	~ Constant (1.3, ,)	mg/min
4: Q	~ Uniform (2.5, 10,)	ACH
5: V	~ Uniform (1200, 1470,)	m^3
6: Vn	~ Triangular (3, 1, 8)	m^3
7: ß	~ Lognormal (5.588, 1.477,)	m^3/min
8: tg	~ Constant (2, ,)	min
9: t1	~ Constant (0, ,)	min
10: t2	~ Constant (9, ,)	min

TASK 3 - Break

Description:

Model: M201

Model Name: 2Box.CE.Gv

Variable	Variable Model	Units
1: Frequency	~ ConstantInt (1, ,)	/shift
2: Duration	~ UniformInt (18, 19,)	min
3: G	~ Constant (0, ,)	mg/min
4: Q	~ Uniform (2.5, 10,)	ACH
5: V	~ Uniform (1200, 1470,)	m^3
6: Vn	~ Triangular (3, 1, 8)	m^3
7: ß	~ Lognormal (5.588, 1.477,)	m^3/min
8: tg	~ Constant (0, ,)	min
9: t1	~ Constant (0, ,)	min
10: t2	~ Constant (19, ,)	min

TASK 4 - T2

Description:

Model: M201

Model Name: 2Box.CE.Gv

Variable	Variable Model	Units
1: Frequency	~ ConstantInt (4, ,)	/shift
2: Duration	~ UniformInt (7, 9,)	min
3: G	~ Constant (2, ,)	mg/min
4: Q	~ Uniform (2.5, 10,)	ACH
5: V	~ Uniform (1200, 1470,)	m^3
6: Vn	~ Triangular (3, 1, 8)	m^3
7: ß	~ Lognormal (5.588, 1.477,)	m^3/min
8: tg	~ Constant (2, ,)	min
9: t1	~ Constant (0, ,)	min
10: t2	~ Constant (9, ,)	min

TASK 5 - Break

Description:

Model: M201

Model Name: 2Box.CE.Gv

Variable	Variable Model	Units
1: Frequency	~ ConstantInt (1, ,)	/shift
2: Duration	~ UniformInt (18, 19,)	min
3: G	~ Constant (0, ,)	mg/min
4: Q	~ Uniform (2.5, 10,)	ACH
5: V	~ Uniform (1200, 1470,)	m^3
6: Vn	~ Triangular (3, 1, 8)	m^3
7: ß	~ Lognormal (5.588, 1.477,)	m^3/min
8: tg	~ Constant (0, ,)	min
9: t1	~ Constant (0, ,)	min
10: t2	~ Constant (19, ,)	min

TASK 6 - T3

Description:

Model: M201

Model Name: 2Box.CE.Gv

Variable	Variable Model	Units
1: Frequency	~ ConstantInt (4, ,)	/shift
2: Duration	~ UniformInt (7, 9,)	min
3: G	~ Constant (5.9, ,)	mg/min
4: Q	~ Uniform (2.5, 10,)	ACH
5: V	~ Uniform (1200, 1470,)	m^3
6: Vn	~ Triangular (3, 1, 8)	m^3
7: ß	~ Lognormal (5.588, 1.477,)	m^3/min
8: tg	~ Uniform (2, 3,)	min
9: t1	~ Constant (0, ,)	min
10: t2	~ Constant (9, ,)	min

TASK 7 - Break

Description:

Model: M201

Model Name: 2Box.CE.Gv

Variable	Variable Model	Units
1: Frequency	~ ConstantInt (1, ,)	/shift
2: Duration	~ UniformInt (18, 19,)	min
3: G	~ Constant (0, ,)	mg/min
4: Q	~ Uniform (2.5, 10,)	ACH
5: V	~ Uniform (1200, 1470,)	m^3
6: Vn	~ Triangular (3, 1, 8)	m^3
7: ß	~ Lognormal (5.588, 1.477,)	m^3/min
8: tg	~ Constant (0, ,)	min
9: t1	~ Constant (0, ,)	min
10: t2	~ Constant (19, ,)	min

TASK 8 - T4

Description:

Model: M201

Model Name: 2Box.CE.Gv

Variable	Variable Model	Units
1: Frequency	~ ConstantInt (4, ,)	/shift
2: Duration	~ UniformInt (7, 9,)	min
3: G	~ Constant (0.9, ,)	mg/min
4: Q	~ Uniform (2.5, 10,)	ACH
5: V	~ Uniform (1200, 1470,)	m^3
6: Vn	~ Triangular (3, 1, 8)	m^3
7: ß	~ Lognormal (5.588, 1.477,)	m^3/min
8: tg	~ Constant (2, ,)	min
9: t1	~ Constant (0, ,)	min
10: t2	~ Constant (9, ,)	min

TASK 9 - Break

Description:

Model: M201

Model Name: 2Box.CE.Gv

Variable	Variable Model	Units
1: Frequency	~ ConstantInt (1, ,)	/shift
2: Duration	~ UniformInt (18, 19,)	min
3: G	~ Constant (0, ,)	mg/min
4: Q	~ Uniform (2.5, 10,)	ACH
5: V	~ Uniform (1200, 1470,)	m^3
6: Vn	~ Triangular (3, 1, 8)	m^3
7: ß	~ Lognormal (5.588, 1.477,)	m^3/min

8: tg	~ Constant (0, ,)	min
9: t1	~ Constant (0, ,)	min
10: t2	~ Constant (19, ,)	min

TASK 10 - T5

Description:

Model: M201

Model Name: 2Box.CE.Gv

**Variable**	**Variable Model**	**Units**
1: Frequency	~ ConstantInt (4, ,)	/shift
2: Duration	~ UniformInt (7, 9,)	min
3: G	~ Constant (1.6, ,)	mg/min
4: Q	~ Uniform (2.5, 10,)	ACH
5: V	~ Uniform (1200, 1470,)	m^3
6: Vn	~ Triangular (3, 1, 8)	m^3
7: ß	~ Lognormal (5.588, 1.477,)	m^3/min
8: tg	~ Constant (2, ,)	min
9: t1	~ Constant (0, ,)	min
10: t2	~ Constant (9, ,)	min

TASK 11 - Break

Description:

Model: M201

Model Name: 2Box.CE.Gv

**Variable**	**Variable Model**	**Units**
1: Frequency	~ ConstantInt (1, ,)	/shift
2: Duration	~ UniformInt (18, 19,)	min
3: G	~ Constant (0, ,)	mg/min
4: Q	~ Uniform (2.5, 10,)	ACH
5: V	~ Uniform (1200, 1470,)	m^3
6: Vn	~ Triangular (3, 1, 8)	m^3
7: ß	~ Lognormal (5.588, 1.477,)	m^3/min
8: tg	~ Constant (0, ,)	min
9: t1	~ Constant (0, ,)	min
10: t2	~ Constant (19, ,)	min

TASK 12 - T6

Description:

Model: M201

Model Name: 2Box.CE.Gv

**Variable**	**Variable Model**	**Units**
1: Frequency	~ ConstantInt (4, ,)	/shift
2: Duration	~ UniformInt (7, 9,)	min

3: G	~ Constant (4.5, ,)	mg/min
4: Q	~ Uniform (2.5, 10,)	ACH
5: V	~ Uniform (1200, 1470,)	m^3
6: Vn	~ Triangular (3, 1, 8)	m^3
7: ß	~ Lognormal (5.588, 1.477,)	m^3/min
8: tg	~ Uniform (2, 3,)	min
9: t1	~ Constant (0, ,)	min
10: t2	~ Constant (19, ,)	min

TASK 13 - Break

Description:

Model: M201

Model Name: 2Box.CE.Gv

Variable	Variable Model	Units
1: Frequency	~ ConstantInt (1, ,)	/shift
2: Duration	~ UniformInt (18, 19,)	min
3: G	~ Constant (0, ,)	mg/min
4: Q	~ Uniform (2.5, 10,)	ACH
5: V	~ Uniform (1200, 1470,)	m^3
6: Vn	~ Triangular (3, 1, 8)	m^3
7: ß	~ Lognormal (5.588, 1.477,)	m^3/min
8: tg	~ Constant (0, ,)	min
9: t1	~ Constant (0, ,)	min
10: t2	~ Constant (19, ,)	min

TWA CALCULATION (TASK OR FULL SHIFT)

Task TWA - the TWA is calculated for the duration of the task(s).

Comments:

SIMULATION OPTIONS

Unless noted below, for all variables modeled with a statistical distribution a random value was generated for each repeat of a task.

Room Variables:
- For each TWA, the room ventilation rate Q generated for the first Box model task was used for all of the following Box model tasks.
- For each TWA, the room volume V generated for the first Box model task was used for all of the following Box model tasks.

Options for 2Box variables t1, t2, and tg:
- If a random t1, t2 or tg > 0, apply following rules:
  1. if tg > Duration, let tg = Duration
  2. if t2 > Duration, let t2 = Duration
  3. if t1 > t2, let t1=1/2 t2

Options for 2Box Constant Emission Model variables t2 and tg:
- tg is not linked to task Duration (DEFAULT)
- t2 is always equal to task Duration (DEFAULT)

STATISTICS

Output Units: mg/m3
Simulation N: 10000

Non-parametric Statistics
Min: 0.064
Max: 0.145
Median: 0.093
Mode: 0.095

X0.05: 0.079
X0.50: 0.093
X0.60: 0.096
X0.70: 0.099
X0.75: 0.100
X0.80: 0.102
X0.90: 0.107
X0.95: 0.111
X0.99: 0.120
X0.999: 0.131

Normal Distribution Statistics

Mean:	0.094
SD:	0.010

Lognormal Distribution Statistics

GM:	0.093
GSD:	1.111

X0.95:	0.111
X0.99:	0.119

GRAPHS

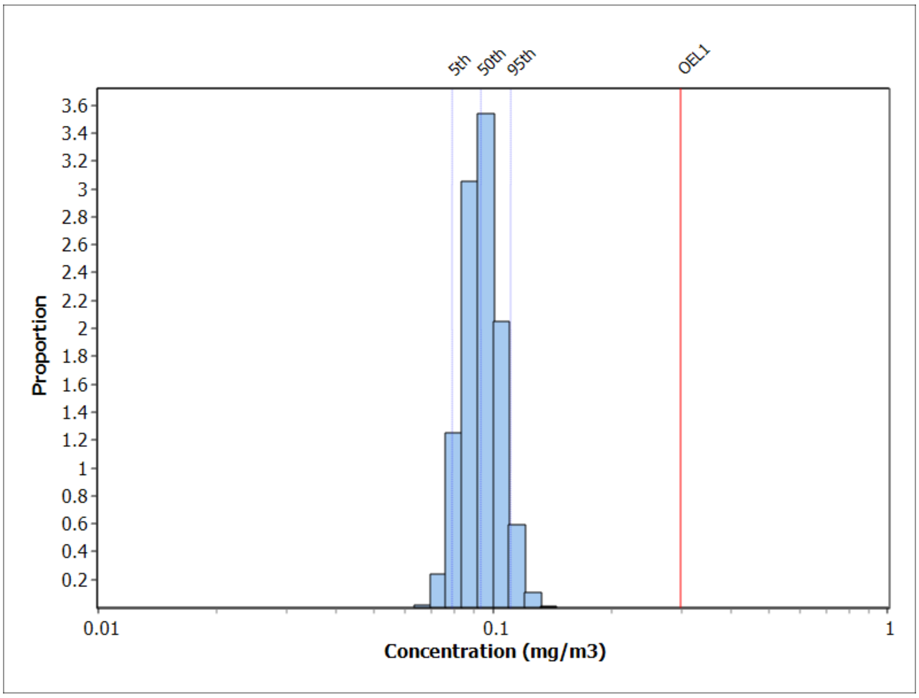

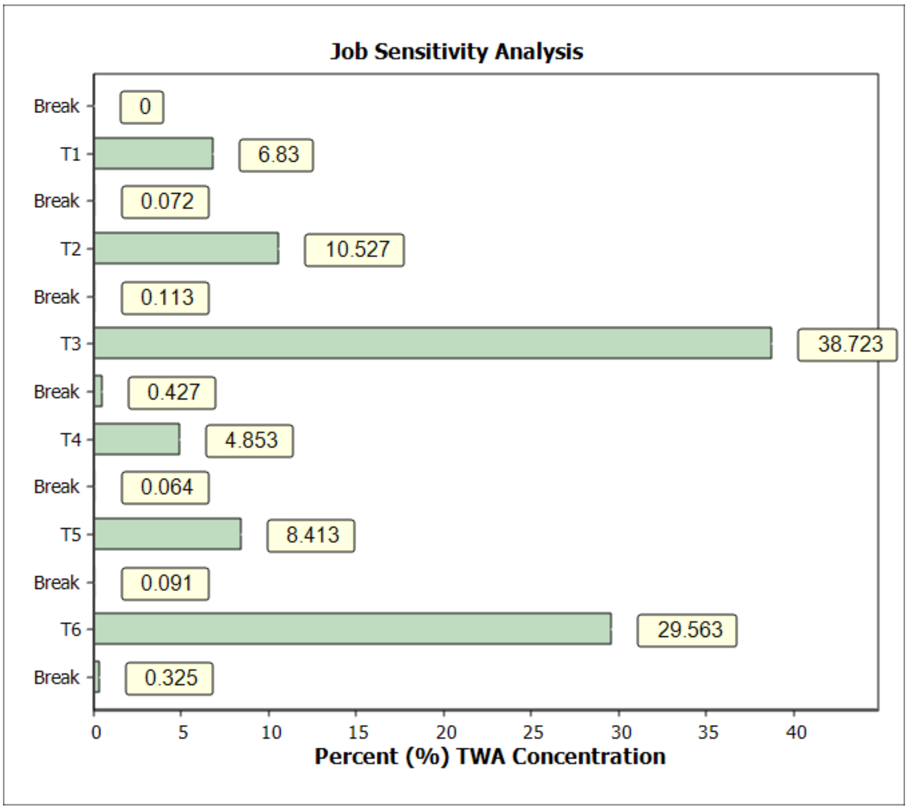
Job Sensitivity Analysis
Break
T1
T2
T3
T4
T5
T6
0
6.83
0.072
10.527
0.113
38.723
0.427
4.853
0.064
8.413
0.091
29.563
0.325
0 5 10 15 20 25 30 35 40
Percent (%) TWA Concentration

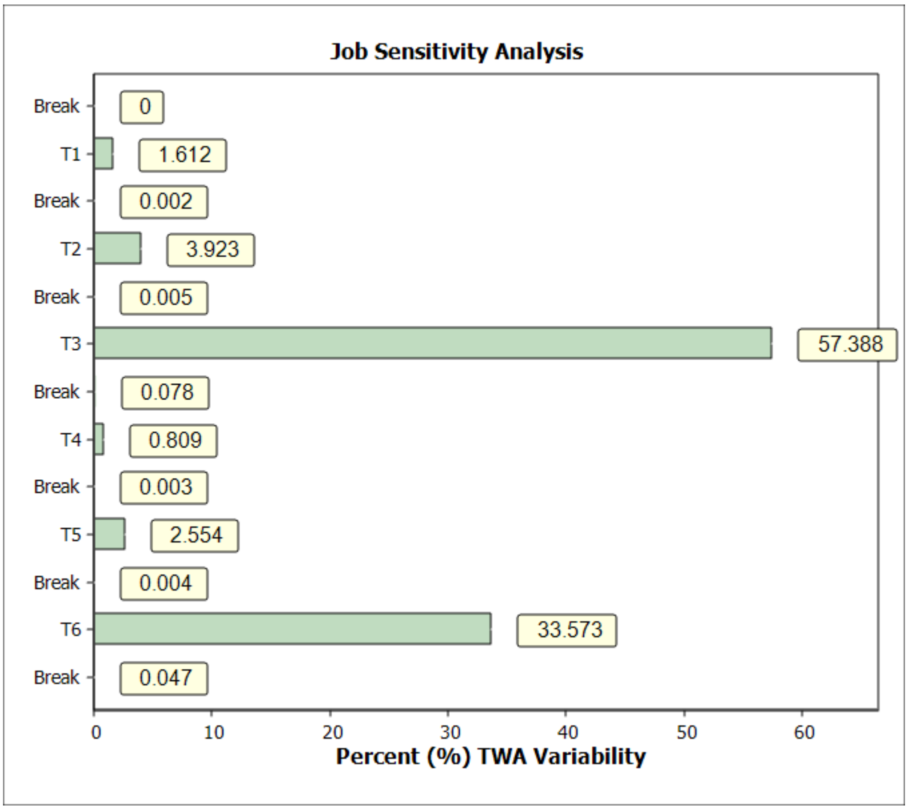
Job Sensitivity Analysis
Break
T1
T2
T3
T4
T5
T6
0
1.612
0.002
3.923
0.005
57.388
0.078
0.809
0.003
2.554
0.004
33.573
0.047
0 10 20 30 40 50 60
Percent (%) TWA Variability

TEXT S4: TiO_2 Far-Field

Version: TEAS 2019 (Version 1.0)
Date: 6/24/2021 3:20:14 PM

SUBSTANCE INFORMATION

TiO_2-N particles dispersed in EtOH (1% w/w). TiO_2 content of aerosol particles at NF was 44% according to the ICP-MS analysis of collected particles.

EXPOSURE LIMITS

Limit value	Type	Source	Notes
REL: 0.3 mg/m3	TWA	NIOSH REL	Here concentrations are for total dust (44% is TiO_2 from the total dust)

EXPOSURE LIMITS

	Type	Source	Notes
OEL1: 0.3 ug/m3	TWA	NIOSH REL	

TASK INFORMATION

TASK 1 - T1
Description:
Model: M201
Model Name: 2Box.CE.Gv

Variable	Variable Model	Units
1: Frequency	~ ConstantInt (4, ,)	/shift
2: Duration	~ UniformInt (7, 9,)	min
3: G	~ Constant (1.3, ,)	mg/min
4: Q	~ Uniform (2.5, 10,)	ACH
5: V	~ Uniform (1200, 1470,)	m^3
6: Vn	~ Triangular (3, 1, 8)	m^3
7: ß	~ Lognormal (5.588, 1.477,)	m^3/min
8: tg	~ Constant (2, ,)	min
9: t1	~ Constant (0, ,)	min
10: t2	~ Constant (0, ,)	min

TASK 2 - Break
Description:
Model: M201
Model Name: 2Box.CE.Gv

Variable	Variable Model	Units
1: Frequency	~ ConstantInt (1, ,)	/shift
2: Duration	~ UniformInt (18, 19,)	min
3: G	~ Constant (0, ,)	mg/min
4: Q	~ Uniform (2.5, 10,)	ACH
5: V	~ Uniform (1200, 1470,)	m^3
6: Vn	~ Triangular (3, 1, 8)	m^3
7: ß	~ Lognormal (5.588, 1.477,)	m^3/min
8: tg	~ Constant (0, ,)	min
9: t1	~ Constant (0, ,)	min
10: t2	~ Constant (0, ,)	min

TASK 3 - T2
Description:
Model: M201
Model Name: 2Box.CE.Gv

Variable	Variable Model	Units
1: Frequency	~ ConstantInt (4, ,)	/shift
2: Duration	~ UniformInt (7, 9,)	min
3: G	~ Constant (2, ,)	mg/min
4: Q	~ Uniform (2.5, 10,)	ACH
5: V	~ Uniform (1200, 1470,)	m^3
6: Vn	~ Triangular (3, 1, 8)	m^3
7: ß	~ Lognormal (5.588, 1.477,)	m^3/min
8: tg	~ Constant (2, ,)	min
9: t1	~ Constant (0, ,)	min
10: t2	~ Constant (0, ,)	min

TASK 4 - Break

Description:

Model: M201

Model Name: 2Box.CE.Gv

Variable	Variable Model	Units
1: Frequency	~ ConstantInt (1, ,)	/shift
2: Duration	~ UniformInt (18, 19,)	min
3: G	~ Constant (0, ,)	mg/min
4: Q	~ Uniform (2.5, 10,)	ACH
5: V	~ Uniform (1200, 1470,)	m^3
6: Vn	~ Triangular (3, 1, 8)	m^3
7: ß	~ Lognormal (5.588, 1.477,)	m^3/min
8: tg	~ Constant (0, ,)	min
9: t1	~ Constant (0, ,)	min
10: t2	~ Constant (0, ,)	min

TASK 5 - T3

Description:

Model: M201

Model Name: 2Box.CE.Gv

Variable	Variable Model	Units
1: Frequency	~ ConstantInt (4, ,)	/shift
2: Duration	~ UniformInt (7, 9,)	min
3: G	~ Constant (5.9, ,)	mg/min
4: Q	~ Uniform (2.5, 10,)	ACH
5: V	~ Uniform (1200, 1470,)	m^3
6: Vn	~ Triangular (3, 1, 8)	m^3
7: ß	~ Lognormal (5.588, 1.477,)	m^3/min
8: tg	~ Uniform (2, 3,)	min
9: t1	~ Constant (0, ,)	min
10: t2	~ Constant (0, ,)	min

TASK 6 - Break

Description:

Model: M201

Model Name: 2Box.CE.Gv

Variable	Variable Model	Units
1: Frequency	~ ConstantInt (1, ,)	/shift
2: Duration	~ UniformInt (18, 19,)	min
3: G	~ Constant (0, ,)	mg/min
4: Q	~ Uniform (2.5, 10,)	ACH
5: V	~ Uniform (1200, 1470,)	m^3
6: Vn	~ Triangular (3, 1, 8)	m^3
7: ß	~ Lognormal (5.588, 1.477,)	m^3/min

8: tg	~ Constant (0, ,)	min
9: t1	~ Constant (0, ,)	min
10: t2	~ Constant (0, ,)	min

TASK 7 - T4

Description:

Model: M201

Model Name: 2Box.CE.Gv

**Variable**	**Variable Model**	**Units**
1: Frequency	~ ConstantInt (4, ,)	/shift
2: Duration	~ UniformInt (7, 9,)	min
3: G	~ Constant (0.9, ,)	mg/min
4: Q	~ Uniform (2.5, 10,)	ACH
5: V	~ Uniform (1200, 1470,)	m^3
6: Vn	~ Triangular (3, 1, 8)	m^3
7: ß	~ Lognormal (5.588, 1.477,)	m^3/min
8: tg	~ Constant (2, ,)	min
9: t1	~ Constant (0, ,)	min
10: t2	~ Constant (0, ,)	min

TASK 8 - Break

Description:

Model: M201

Model Name: 2Box.CE.Gv

**Variable**	**Variable Model**	**Units**
1: Frequency	~ ConstantInt (1, ,)	/shift
2: Duration	~ UniformInt (18, 19,)	min
3: G	~ Constant (0, ,)	mg/min
4: Q	~ Uniform (2.5, 10,)	ACH
5: V	~ Uniform (1200, 1470,)	m^3
6: Vn	~ Triangular (3, 1, 8)	m^3
7: ß	~ Lognormal (5.588, 1.477,)	m^3/min
8: tg	~ Constant (0, ,)	min
9: t1	~ Constant (0, ,)	min
10: t2	~ Constant (0, ,)	min

TASK 9 - T5

Description:

Model: M201

Model Name: 2Box.CE.Gv

**Variable**	**Variable Model**	**Units**
1: Frequency	~ ConstantInt (4, ,)	/shift
2: Duration	~ UniformInt (7, 9,)	min

3: G	~ Constant (1.6, ,)	mg/min
4: Q	~ Uniform (2.5, 10,)	ACH
5: V	~ Uniform (1200, 1470,)	m^3
6: Vn	~ Triangular (3, 1, 8)	m^3
7: ß	~ Lognormal (5.588, 1.477,)	m^3/min
8: tg	~ Constant (2, ,)	min
9: t1	~ Constant (0, ,)	min
10: t2	~ Constant (0, ,)	min

TASK 10 - Break

Description:

Model: M201

Model Name: 2Box.CE.Gv

**Variable**	**Variable Model**	**Units**
1: Frequency	~ ConstantInt (1, ,)	/shift
2: Duration	~ UniformInt (18, 19,)	min
3: G	~ Constant (0, ,)	mg/min
4: Q	~ Uniform (2.5, 10,)	ACH
5: V	~ Uniform (1200, 1470,)	m^3
6: Vn	~ Triangular (3, 1, 8)	m^3
7: ß	~ Lognormal (5.588, 1.477,)	m^3/min
8: tg	~ Constant (0, ,)	min
9: t1	~ Constant (0, ,)	min
10: t2	~ Constant (0, ,)	min

TASK 11 - T6

Description:

Model: M201

Model Name: 2Box.CE.Gv

**Variable**	**Variable Model**	**Units**
1: Frequency	~ ConstantInt (4, ,)	/shift
2: Duration	~ UniformInt (7, 9,)	min
3: G	~ Constant (4.5, ,)	mg/min
4: Q	~ Uniform (2.5, 10,)	ACH
5: V	~ Uniform (1200, 1470,)	m^3
6: Vn	~ Triangular (3, 1, 8)	m^3
7: ß	~ Lognormal (5.588, 1.477,)	m^3/min
8: tg	~ Uniform (2, 3,)	min
9: t1	~ Constant (0, ,)	min
10: t2	~ Constant (0, ,)	min

TASK 12 - Break

Description:

Model: M201

Model Name: 2Box.CE.Gv

Variable	Variable Model	Units
1: Frequency	~ ConstantInt (1, ,)	/shift
2: Duration	~ UniformInt (18, 19,)	min
3: G	~ Constant (0, ,)	mg/min
4: Q	~ Uniform (2.5, 10,)	ACH
5: V	~ Uniform (1200, 1470,)	m^3
6: Vn	~ Triangular (3, 1, 8)	m^3
7: ß	~ Lognormal (5.588, 1.477,)	m^3/min
8: tg	~ Constant (0, ,)	min
9: t1	~ Constant (0, ,)	min
10: t2	~ Constant (0, ,)	min

TWA CALCULATION (TASK OR FULL SHIFT)

Task TWA - the TWA is calculated for the duration of the task(s).

SIMULATION OPTIONS

Unless noted below, for all variables modeled with a statistical distribution a random value was generated for each repeat of a task.

Room Variables:
- For each TWA, the room ventilation rate Q generated for the first Box model task was used for all of the following Box model tasks.
- For each TWA, the room volume V generated for the first Box model task was used for all of the following Box model tasks.

Options for 2Box variables t1, t2, and tg:
- If a random t1, t2 or tg > 0, apply following rules:
  1. if tg > Duration, let tg = Duration
  2. if t2 > Duration, let t2 = Duration
  3. if t1 > t2, let t1=1/2 t2

Options for 2Box Constant Emission Model variables t2 and tg:
- tg is not linked to task Duration (DEFAULT)
- t2 is always equal to task Duration (DEFAULT)

STATISTICS

Output Units: mg/m3

Simulation N: 10000

Non-parametric Statistics
Min: 0.002
Max: 0.010
Median: 0.004
Mode: 0.003

X0.05: 0.002
X0.50: 0.004
X0.60: 0.004
X0.70: 0.005
X0.75: 0.005
X0.80: 0.005
X0.90: 0.007
X0.95: 0.007
X0.99: 0.008
X0.999: 0.009

Normal Distribution Statistics
Mean: 0.004
SD: 0.002

Lognormal Distribution Statistics
GM: 0.004
GSD: 1.452

X0.95: 0.007
X0.99: 0.009

GRAPHS

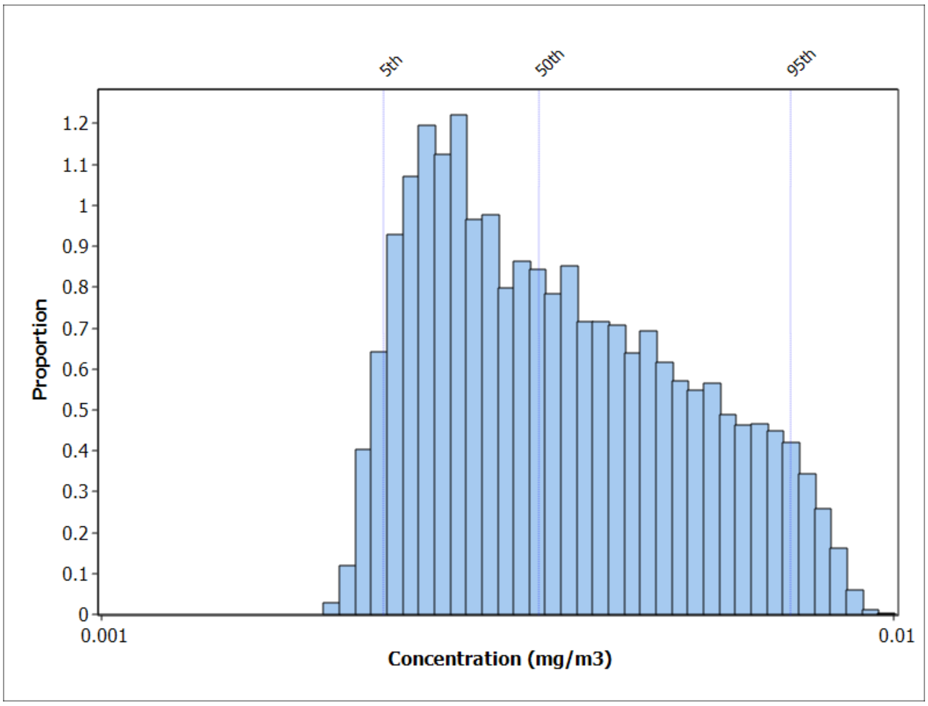
5th
50th
95th
Proportion
1.2
1.1
1
0.9
0.8
0.7
0.6
0.5
0.4
0.3
0.2
0.1
0
0.001
0.01
Concentration (mg/m3)

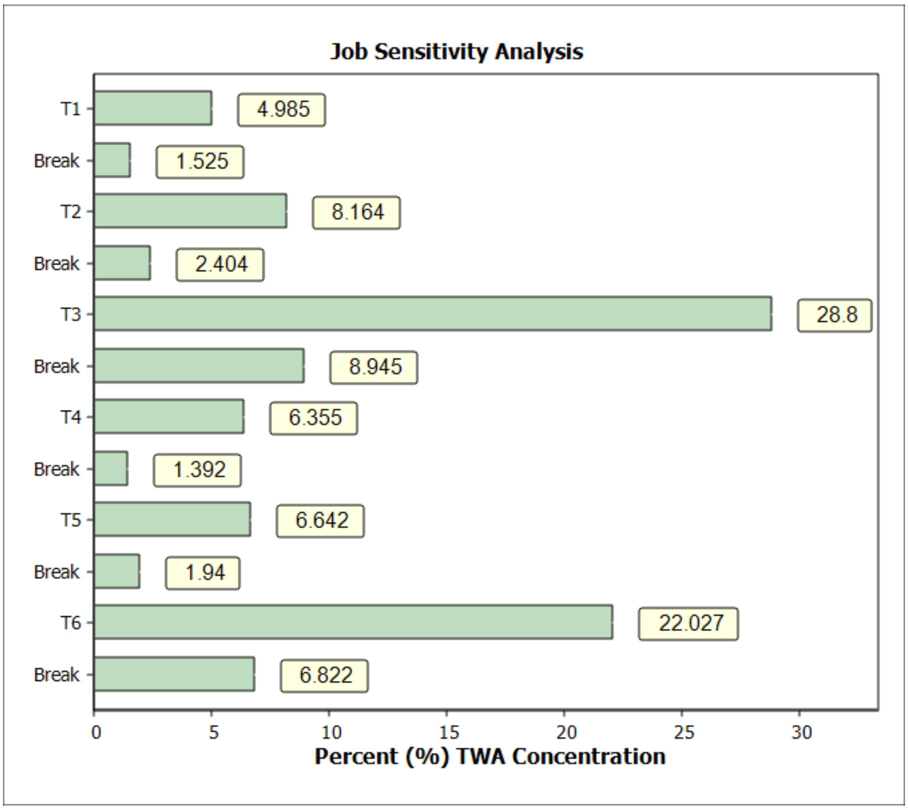
Job Sensitivity Analysis
T1
Break
T2
Break
T3
Break
T4
Break
T5
Break
T6
Break
4.985
1.525
8.164
2.404
28.8
8.945
6.355
1.392
6.642
1.94
22.027
6.822
0
5
10
15
20
25
30
Percent (%) TWA Concentration

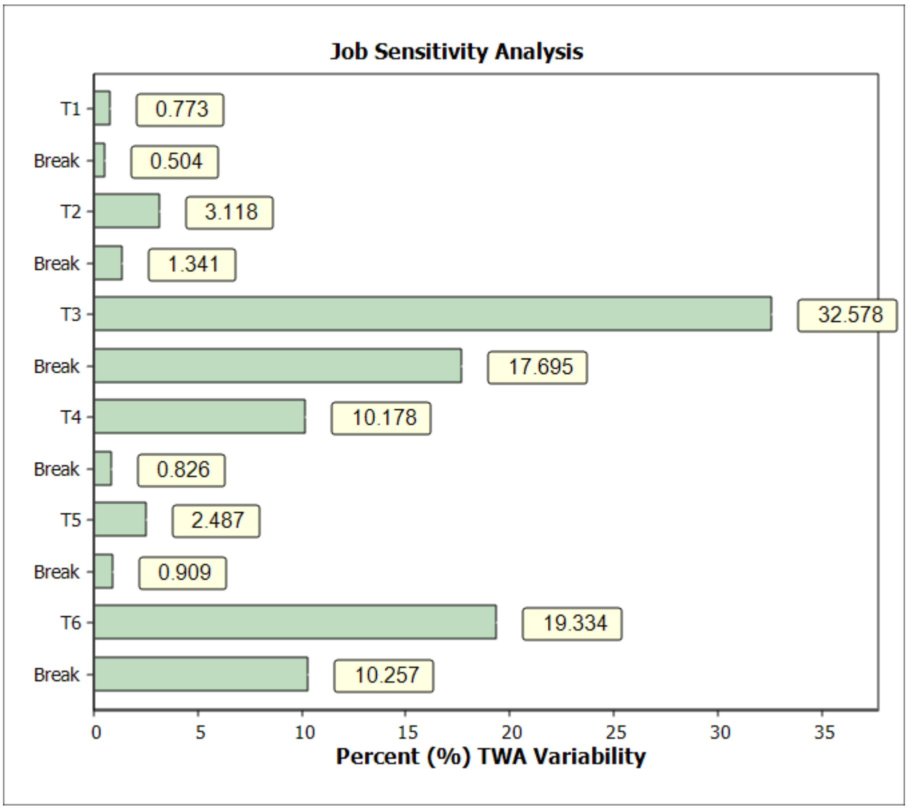
Job Sensitivity Analysis
T1
Break
T2
Break
T3
Break
T4
Break
T5
Break
T6
Break
0.773
0.504
3.118
1.341
32.578
17.695
10.178
0.826
2.487
0.909
19.334
10.257
0
5
10
15
20
25
30
35
Percent (%) TWA Variability

TEXT S5: Ag-HEC Near-Field

Version: TEAS 2019 (Version 1.0)
Date: 6/24/2021 3:26:46 PM

SUBSTANCE INFORMATION

Ag-HEC particles dispersed in water. Ag content of aerosol particles at NF was 1.1% according to the ICP-MS analysis of collected particles.

EXPOSURE LIMITS

Limit value	Type	Source	Notes
REL: 0.9 ug/m3	TWA	NIOSH REL	Here concentrations are for total dust (1.1% is Ag from the total dust)

TASK INFORMATION

TASK 1 - T7
Description:
Model: M201
Model Name: 2Box.CE.Gv

Variable	Variable Model	Units
1: Frequency	~ ConstantInt (4, ,)	/shift
2: Duration	~ ConstantInt (11, ,)	min
3: G	~ Constant (0, ,)	mg/min
4: Q	~ Uniform (2.5, 10,)	ACH
5: V	~ Uniform (1200, 1470,)	m^3
6: Vn	~ Triangular (3, 1, 8)	m^3
7: ß	~ Lognormal (5.588, 1.477,)	m^3/min
8: tg	~ Constant (0, ,)	min
9: t1	~ Constant (0, ,)	min
10: t2	~ Constant (11, ,)	min

TASK 2 - Break
Description:
Model: M201
Model Name: 2Box.CE.Gv

Variable	Variable Model	Units
1: Frequency	~ ConstantInt (1, ,)	/shift
2: Duration	~ ConstantInt (19, ,)	min
3: G	~ Constant (0, ,)	mg/min
4: Q	~ Uniform (2.5, 10,)	ACH
5: V	~ Uniform (1200, 1470,)	m^3
6: Vn	~ Triangular (3, 1, 8)	m^3
7: ß	~ Lognormal (5.588, 1.477,)	m^3/min
8: tg	~ Constant (0, ,)	min
9: t1	~ Constant (0, ,)	min
10: t2	~ Constant (19, ,)	min

TASK 3 - T8
Description:
Model: M201
Model Name: 2Box.CE.Gv

Variable	Variable Model	Units
1: Frequency	~ ConstantInt (4, ,)	/shift
2: Duration	~ UniformInt (7, 9,)	min
3: G	~ Constant (0.8, ,)	mg/min
4: Q	~ Uniform (2.5, 10,)	ACH
5: V	~ Uniform (1200, 1470,)	m^3
6: Vn	~ Triangular (3, 1, 8)	m^3
7: ß	~ Lognormal (5.588, 1.477,)	m^3/min
8: tg	~ Uniform (1, 2,)	min
9: t1	~ Constant (0, ,)	min
10: t2	~ Constant (9, ,)	min

TASK 4 - Break
Description:
Model: M201
Model Name: 2Box.CE.Gv

Variable	Variable Model	Units
1: Frequency	~ ConstantInt (1, ,)	/shift
2: Duration	~ ConstantInt (19, ,)	min
3: G	~ Constant (0, ,)	mg/min
4: Q	~ Uniform (2.5, 10,)	ACH
5: V	~ Uniform (1200, 1470,)	m^3
6: Vn	~ Triangular (3, 1, 8)	m^3

7: ß	~ Lognormal (5.588, 1.477,)	m³/min
8: tg	~ Constant (0, ,)	min
9: t1	~ Constant (0, ,)	min
10: t2	~ Constant (19, ,)	min

TASK 5 - T9

Description:

Model: M201

Model Name: 2Box.CE.Gv

Variable	Variable Model	Units
1: Frequency	~ ConstantInt (4, ,)	/shift
2: Duration	~ UniformInt (7, 9,)	min
3: G	~ Constant (1.3, ,)	mg/min
4: Q	~ Uniform (2.5, 10,)	ACH
5: V	~ Uniform (1200, 1470,)	m³
6: Vn	~ Triangular (3, 1, 8)	m³
7: ß	~ Lognormal (5.588, 1.477,)	m³/min
8: tg	~ Uniform (1, 2,)	min
9: t1	~ Constant (0, ,)	min
10: t2	~ Constant (9, ,)	min

TASK 6 - Break

Description:

Model: M201

Model Name: 2Box.CE.Gv

Variable	Variable Model	Units
1: Frequency	~ ConstantInt (1, ,)	/shift
2: Duration	~ ConstantInt (19, ,)	min
3: G	~ Constant (0, ,)	mg/min
4: Q	~ Uniform (2.5, 10,)	ACH
5: V	~ Uniform (1200, 1470,)	m³
6: Vn	~ Triangular (3, 1, 8)	m³
7: ß	~ Lognormal (5.588, 1.477,)	m³/min
8: tg	~ Constant (0, ,)	min
9: t1	~ Constant (0, ,)	min
10: t2	~ Constant (19, ,)	min

TASK 7 - T10

Description:

Model: M201

Model Name: 2Box.CE.Gv

Variable	Variable Model	Units
1: Frequency	~ ConstantInt (4, ,)	/shift
2: Duration	~ UniformInt (7, 9,)	min
3: G	~ Constant (1.9, ,)	mg/min
4: Q	~ Uniform (2.5, 10,)	ACH
5: V	~ Uniform (1200, 1470,)	m^3
6: Vn	~ Triangular (3, 1, 8)	m^3
7: ß	~ Lognormal (5.588, 1.477,)	m^3/min
8: tg	~ Constant (2, ,)	min
9: t1	~ Constant (0, ,)	min
10: t2	~ Constant (19, ,)	min

TASK 8 - Break

Description:

Model: M201

Model Name: 2Box.CE.Gv

Variable	Variable Model	Units
1: Frequency	~ ConstantInt (1, ,)	/shift
2: Duration	~ ConstantInt (19, ,)	min
3: G	~ Constant (0, ,)	mg/min
4: Q	~ Uniform (2.5, 10,)	ACH
5: V	~ Uniform (1200, 1470,)	m^3
6: Vn	~ Triangular (3, 1, 8)	m^3
7: ß	~ Lognormal (5.588, 1.477,)	m^3/min
8: tg	~ Constant (0, ,)	min
9: t1	~ Constant (0, ,)	min
10: t2	~ Constant (19, ,)	min

TASK 9 - T11

Description:

Model: M201

Model Name: 2Box.CE.Gv

Variable	Variable Model	Units
1: Frequency	~ ConstantInt (4, ,)	/shift
2: Duration	~ UniformInt (7, 9,)	min
3: G	~ Constant (2.2, ,)	mg/min
4: Q	~ Uniform (2.5, 10,)	ACH
5: V	~ Uniform (1200, 1470,)	m^3
6: Vn	~ Triangular (3, 1, 8)	m^3
7: ß	~ Lognormal (5.588, 1.477,)	m^3/min
8: tg	~ Uniform (1, 2,)	min
9: t1	~ Constant (0, ,)	min
10: t2	~ Constant (9, ,)	min

TASK 10 - Break
Description:
Model: M201
Model Name: 2Box.CE.Gv

Variable	Variable Model	Units
1: Frequency	~ ConstantInt (1, ,)	/shift
2: Duration	~ ConstantInt (19, ,)	min
3: G	~ Constant (0, ,)	mg/min
4: Q	~ Uniform (2.5, 10,)	ACH
5: V	~ Uniform (1200, 1470,)	m^3
6: Vn	~ Triangular (3, 1, 8)	m^3
7: ß	~ Lognormal (5.588, 1.477,)	m^3/min
8: tg	~ Constant (0, ,)	min
9: t1	~ Constant (0, ,)	min
10: t2	~ Constant (19, ,)	min

TASK 11 - T12
Description:
Model: M201
Model Name: 2Box.CE.Gv

Variable	Variable Model	Units
1: Frequency	~ ConstantInt (4, ,)	/shift
2: Duration	~ UniformInt (7, 9,)	min
3: G	~ Constant (2.6, ,)	mg/min
4: Q	~ Uniform (2.5, 10,)	ACH
5: V	~ Uniform (1200, 1470,)	m^3
6: Vn	~ Triangular (3, 1, 8)	m^3
7: ß	~ Lognormal (5.588, 1.477,)	m^3/min
8: tg	~ Constant (2, ,)	min
9: t1	~ Constant (0, ,)	min
10: t2	~ Constant (19, ,)	min

TASK 12 - Break
Description:
Model: M201
Model Name: 2Box.CE.Gv

Variable	Variable Model	Units
1: Frequency	~ ConstantInt (1, ,)	/shift
2: Duration	~ ConstantInt (19, ,)	min
3: G	~ Constant (0, ,)	mg/min
4: Q	~ Uniform (2.5, 10,)	ACH
5: V	~ Uniform (1200, 1470,)	m^3
6: Vn	~ Triangular (3, 1, 8)	m^3

7: ß	~ Lognormal (5.588, 1.477,)	m^3/min
8: tg	~ Constant (0, ,)	min
9: t1	~ Constant (0, ,)	min
10: t2	~ Constant (19, ,)	min

TWA CALCULATION (TASK OR FULL SHIFT)

Task TWA - the TWA is calculated for the duration of the task(s).

SIMULATION OPTIONS

Unless noted below, for all variables modeled with a statistical distribution a random value was generated for each repeat of a task.

Room Variables:
- For each TWA, the room ventilation rate Q generated for the first Box model task was used for all of the following Box model tasks.
- For each TWA, the room volume V generated for the first Box model task was used for all of the following Box model tasks.

Options for 2Box variables t1, t2, and tg:
- If a random t1, t2 or tg > 0, apply following rules:
  1. if tg > Duration, let tg = Duration
  2. if t2 > Duration, let t2 = Duration
  3. if t1 > t2, let t1=1/2 t2

Options for 2Box Constant Emission Model variables t2 and tg:
- tg is not linked to task Duration (DEFAULT)
- t2 is always equal to task Duration (DEFAULT)

STATISTICS

Output Units: mg/m3
Simulation N: 10000

Non-parametric Statistics
Min: 0.027
Max: 0.060
Median: 0.039
Mode: 0.039

X0.05: 0.033
X0.50: 0.039
X0.60: 0.040

X0.70:	0.041
X0.75:	0.042
X0.80:	0.042
X0.90:	0.044
X0.95:	0.046
X0.99:	0.049
X0.999:	0.053

Normal Distribution Statistics

Mean:	0.039
SD:	0.004

Lognormal Distribution Statistics

GM:	0.039
GSD:	1.106

X0.95:	0.046
X0.99:	0.049

GRAPHS

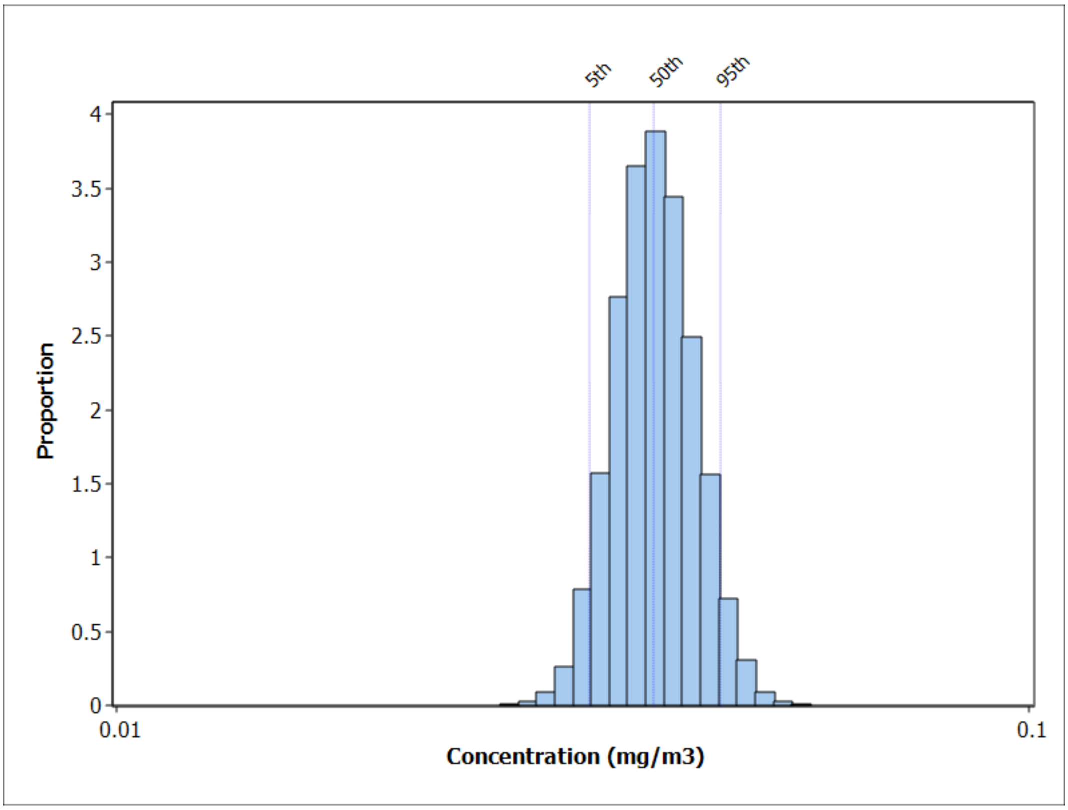
5th
50th
95th
Proportion
4
3.5
3
2.5
2
1.5
1
0.5
0
0.01
0.1
Concentration (mg/m3)

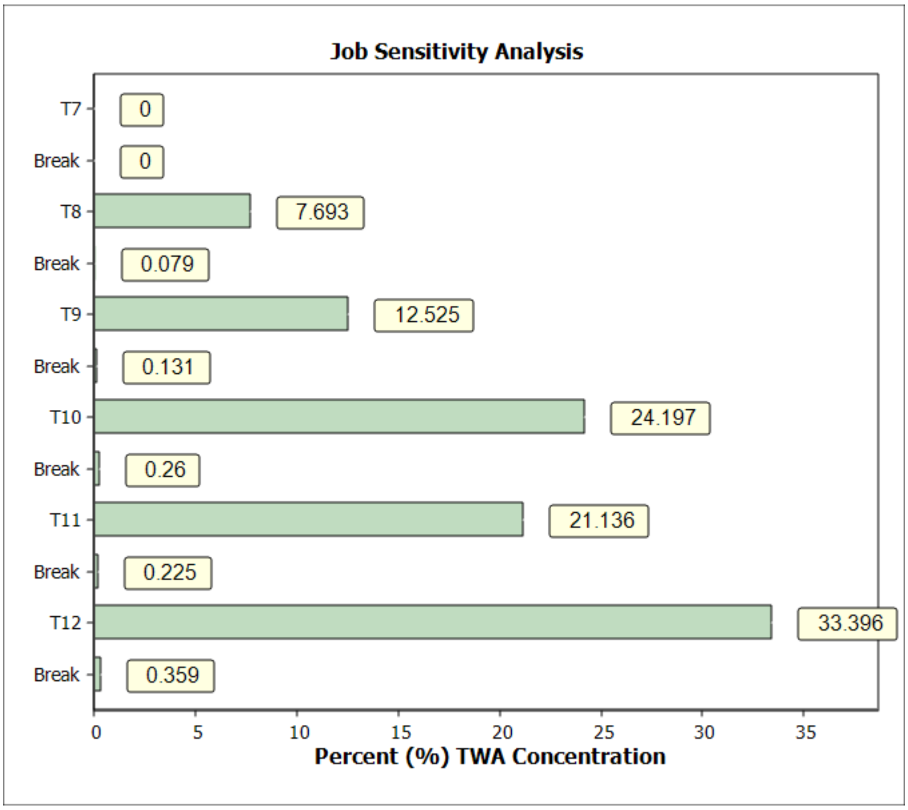
Job Sensitivity Analysis
T7
0
Break
0
T8
7.693
Break
0.079
T9
12.525
Break
0.131
T10
24.197
Break
0.26
T11
21.136
Break
0.225
T12
33.396
Break
0.359
0
5
10
15
20
25
30
35
Percent (%) TWA Concentration

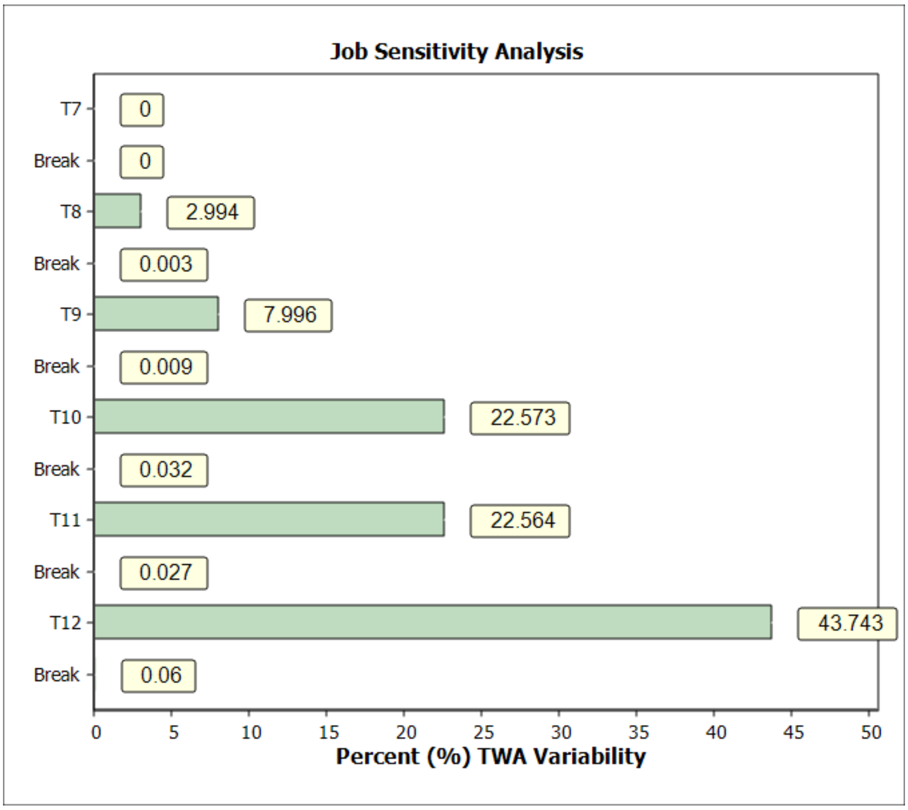
Job Sensitivity Analysis
T7
Break
T8
Break
T9
Break
T10
Break
T11
Break
T12
Break
0
0
2.994
0.003
7.996
0.009
22.573
0.032
22.564
0.027
43.743
0.06
0
5
10
15
20
25
30
35
40
45
50
Percent (%) TWA Variability

TEXT S6: Ag-HEC Far-Field

Version: TEAS 2019 (Version 1.0)

Date: 6/24/2021 3:29:27 PM

SUBSTANCE INFORMATION

Ag-HEC particles dispersed in water. Ag content of aerosol particles at NF was 1.1% according to the ICP-MS analysis of collected particles.

EXPOSURE LIMITS

Limit value	Type	Source	Notes
REL: 0.9 ug/m3	TWA	NIOSH REL	Here concentrations are for total dust (1.1% is Ag from the total dust)

TASK INFORMATION

TASK 1 - T7

Description:

Model: M201

Model Name: 2Box.CE.Gv

Variable	Variable Model	Units
1: Frequency	~ ConstantInt (4, ,)	/shift
2: Duration	~ ConstantInt (11, ,)	min
3: G	~ Constant (0, ,)	mg/min
4: Q	~ Uniform (2.5, 10,)	ACH
5: V	~ Uniform (1200, 1470,)	m^3
6: Vn	~ Triangular (3, 1, 8)	m^3
7: ß	~ Lognormal (5.588, 1.477,)	m^3/min
8: tg	~ Constant (0, ,)	min
9: t1	~ Constant (0, ,)	min
10: t2	~ Constant (0, ,)	min

TASK 2 - Break
Description:
Model: M201
Model Name: 2Box.CE.Gv

Variable	Variable Model	Units
1: Frequency	~ ConstantInt (1, ,)	/shift
2: Duration	~ ConstantInt (19, ,)	min
3: G	~ Constant (0, ,)	mg/min
4: Q	~ Uniform (2.5, 10,)	ACH
5: V	~ Uniform (1200, 1470,)	m^3
6: Vn	~ Triangular (3, 1, 8)	m^3
7: ß	~ Lognormal (5.588, 1.477,)	m^3/min
8: tg	~ Constant (0, ,)	min
9: t1	~ Constant (0, ,)	min
10: t2	~ Constant (0, ,)	min

TASK 3 - T8
Description:
Model: M201
Model Name: 2Box.CE.Gv

Variable	Variable Model	Units
1: Frequency	~ ConstantInt (4, ,)	/shift
2: Duration	~ UniformInt (7, 9,)	min
3: G	~ Constant (0.8, ,)	mg/min
4: Q	~ Uniform (2.5, 10,)	ACH
5: V	~ Uniform (1200, 1470,)	m^3
6: Vn	~ Triangular (3, 1, 8)	m^3
7: ß	~ Lognormal (5.588, 1.477,)	m^3/min
8: tg	~ Uniform (1, 2,)	min
9: t1	~ Constant (0, ,)	min
10: t2	~ Constant (0, ,)	min

TASK 4 - Break
Description:
Model: M201
Model Name: 2Box.CE.Gv

Variable	Variable Model	Units
1: Frequency	~ ConstantInt (1, ,)	/shift
2: Duration	~ ConstantInt (19, ,)	min
3: G	~ Constant (0, ,)	mg/min
4: Q	~ Uniform (2.5, 10,)	ACH
5: V	~ Uniform (1200, 1470,)	m^3
6: Vn	~ Triangular (3, 1, 8)	m^3

7: ß	~ Lognormal (5.588, 1.477,)	m^3/min
8: tg	~ Constant (0, ,)	min
9: t1	~ Constant (0, ,)	min
10: t2	~ Constant (0, ,)	min

TASK 5 - T9

Description:

Model: M201

Model Name: 2Box.CE.Gv

Variable	Variable Model	Units
1: Frequency	~ ConstantInt (4, ,)	/shift
2: Duration	~ UniformInt (7, 9,)	min
3: G	~ Constant (1.3, ,)	mg/min
4: Q	~ Uniform (2.5, 10,)	ACH
5: V	~ Uniform (1200, 1470,)	m^3
6: Vn	~ Triangular (3, 1, 8)	m^3
7: ß	~ Lognormal (5.588, 1.477,)	m^3/min
8: tg	~ Uniform (1, 2,)	min
9: t1	~ Constant (0, ,)	min
10: t2	~ Constant (0, ,)	min

TASK 6 - Break

Description:

Model: M201

Model Name: 2Box.CE.Gv

Variable	Variable Model	Units
1: Frequency	~ ConstantInt (1, ,)	/shift
2: Duration	~ ConstantInt (19, ,)	min
3: G	~ Constant (0, ,)	mg/min
4: Q	~ Uniform (2.5, 10,)	ACH
5: V	~ Uniform (1200, 1470,)	m^3
6: Vn	~ Triangular (3, 1, 8)	m^3
7: ß	~ Lognormal (5.588, 1.477,)	m^3/min
8: tg	~ Constant (0, ,)	min
9: t1	~ Constant (0, ,)	min
10: t2	~ Constant (0, ,)	min

TASK 7 - T10

Description:

Model: M201

Model Name: 2Box.CE.Gv

Variable	Variable Model	Units
1: Frequency	~ ConstantInt (4, ,)	/shift
2: Duration	~ UniformInt (7, 9,)	min
3: G	~ Constant (1.9, ,)	mg/min
4: Q	~ Uniform (2.5, 10,)	ACH
5: V	~ Uniform (1200, 1470,)	m^3
6: Vn	~ Triangular (3, 1, 8)	m^3
7: ß	~ Lognormal (5.588, 1.477,)	m^3/min
8: tg	~ Constant (2, ,)	min
9: t1	~ Constant (0, ,)	min
10: t2	~ Constant (0, ,)	min

TASK 8 - Break

Description:

Model: M201

Model Name: 2Box.CE.Gv

Variable	Variable Model	Units
1: Frequency	~ ConstantInt (1, ,)	/shift
2: Duration	~ ConstantInt (19, ,)	min
3: G	~ Constant (0, ,)	mg/min
4: Q	~ Uniform (2.5, 10,)	ACH
5: V	~ Uniform (1200, 1470,)	m^3
6: Vn	~ Triangular (3, 1, 8)	m^3
7: ß	~ Lognormal (5.588, 1.477,)	m^3/min
8: tg	~ Constant (0, ,)	min
9: t1	~ Constant (0, ,)	min
10: t2	~ Constant (0, ,)	min

TASK 9 - T11

Description:

Model: M201

Model Name: 2Box.CE.Gv

Variable	Variable Model	Units
1: Frequency	~ ConstantInt (4, ,)	/shift
2: Duration	~ UniformInt (7, 9,)	min
3: G	~ Constant (2.2, ,)	mg/min
4: Q	~ Uniform (2.5, 10,)	ACH
5: V	~ Uniform (1200, 1470,)	m^3
6: Vn	~ Triangular (3, 1, 8)	m^3
7: ß	~ Lognormal (5.588, 1.477,)	m^3/min
8: tg	~ Uniform (1, 2,)	min
9: t1	~ Constant (0, ,)	min
10: t2	~ Constant (0, ,)	min

TASK 10 - Break
Description:
Model: M201
Model Name: 2Box.CE.Gv

Variable	Variable Model	Units
1: Frequency	~ ConstantInt (1, ,)	/shift
2: Duration	~ ConstantInt (19, ,)	min
3: G	~ Constant (0, ,)	mg/min
4: Q	~ Uniform (2.5, 10,)	ACH
5: V	~ Uniform (1200, 1470,)	m^3
6: Vn	~ Triangular (3, 1, 8)	m^3
7: ß	~ Lognormal (5.588, 1.477,)	m^3/min
8: tg	~ Constant (0, ,)	min
9: t1	~ Constant (0, ,)	min
10: t2	~ Constant (0, ,)	min

TASK 11 - T12
Description:
Model: M201
Model Name: 2Box.CE.Gv

Variable	Variable Model	Units
1: Frequency	~ ConstantInt (4, ,)	/shift
2: Duration	~ UniformInt (7, 9,)	min
3: G	~ Constant (2.6, ,)	mg/min
4: Q	~ Uniform (2.5, 10,)	ACH
5: V	~ Uniform (1200, 1470,)	m^3
6: Vn	~ Triangular (3, 1, 8)	m^3
7: ß	~ Lognormal (5.588, 1.477,)	m^3/min
8: tg	~ Constant (2, ,)	min
9: t1	~ Constant (0, ,)	min
10: t2	~ Constant (0, ,)	min

TASK 12 - Break
Description:
Model: M201
Model Name: 2Box.CE.Gv

Variable	Variable Model	Units
1: Frequency	~ ConstantInt (1, ,)	/shift
2: Duration	~ ConstantInt (19, ,)	min
3: G	~ Constant (0, ,)	mg/min
4: Q	~ Uniform (2.5, 10,)	ACH
5: V	~ Uniform (1200, 1470,)	m^3
6: Vn	~ Triangular (3, 1, 8)	m^3

7: ß	~ Lognormal (5.588, 1.477,)	m^3/min
8: tg	~ Constant (0, ,)	min
9: t1	~ Constant (0, ,)	min
10: t2	~ Constant (0, ,)	min

TWA CALCULATION (TASK OR FULL SHIFT)

Task TWA - the TWA is calculated for the duration of the task(s).

SIMULATION OPTIONS

Unless noted below, for all variables modeled with a statistical distribution a random value was generated for each repeat of a task.

Room Variables:
- For each TWA, the room ventilation rate Q generated for the first Box model task was used for all of the following Box model tasks.
- For each TWA, the room volume V generated for the first Box model task was used for all of the following Box model tasks.

Options for 2Box variables t1, t2, and tg:
- If a random t1, t2 or tg > 0, apply following rules:
  1. if tg > Duration, let tg = Duration
  2. if t2 > Duration, let t2 = Duration
  3. if t1 > t2, let t1=1/2 t2

Options for 2Box Constant Emission Model variables t2 and tg:
- tg is not linked to task Duration (DEFAULT)
- t2 is always equal to task Duration (DEFAULT)

STATISTICS

Output Units:	μg/m3
Simulation N:	10000
**Non-parametric Statistics**	
Min:	0.749
Max:	3.609
Median:	1.403
Mode:	0.955
X0.05:	0.891
X0.50:	1.403
X0.60:	1.587
X0.70:	1.825
X0.75:	1.961
X0.80:	2.130
X0.90:	2.563
X0.95:	2.847
X0.99:	3.224
X0.999:	3.480
**Normal Distribution Statistics**	
Mean:	1.585
SD:	0.622
**Lognormal Distribution Statistics**	
GM:	1.477
GSD:	1.447
X0.95:	2.712
X0.99:	3.487

GRAPHS

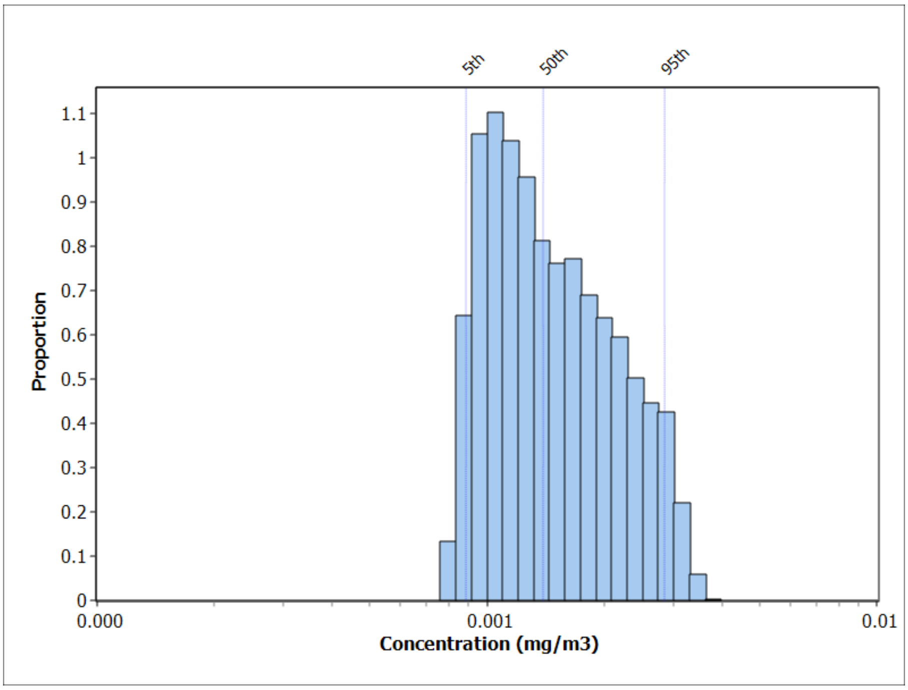
5th
50th
95th
Proportion
1.1
1
0.9
0.8
0.7
0.6
0.5
0.4
0.3
0.2
0.1
0
0.000
0.001
0.01
Concentration (mg/m3)

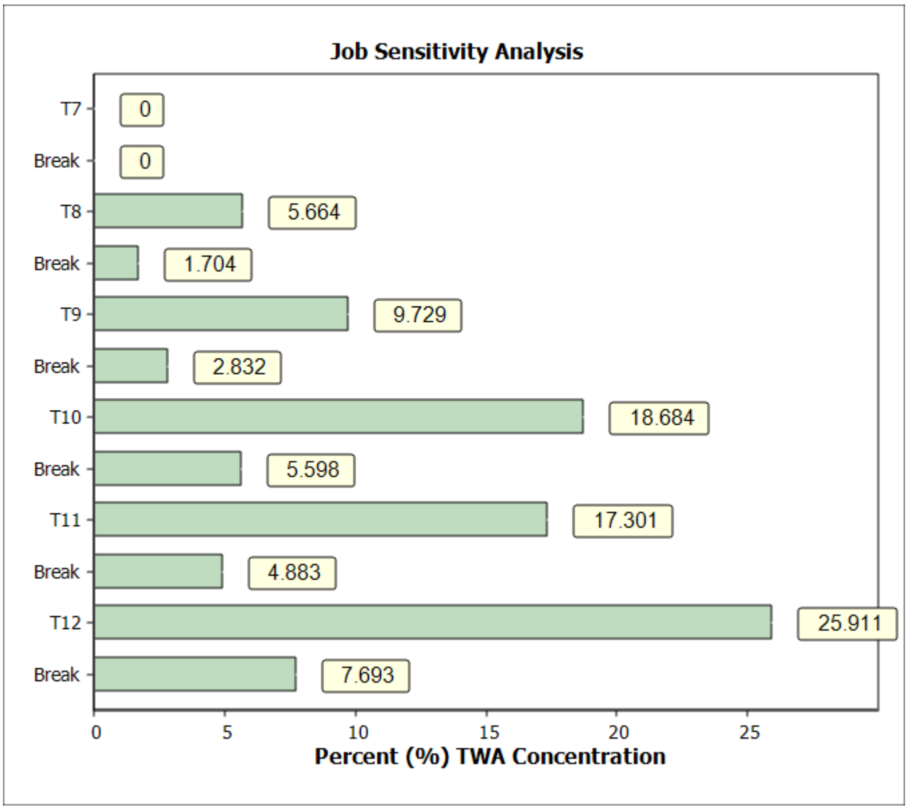
Job Sensitivity Analysis
T7
Break
T8
Break
T9
Break
T10
Break
T11
Break
T12
Break
0
0
5.664
1.704
9.729
2.832
18.684
5.598
17.301
4.883
25.911
7.693
0
5
10
15
20
25
Percent (%) TWA Concentration

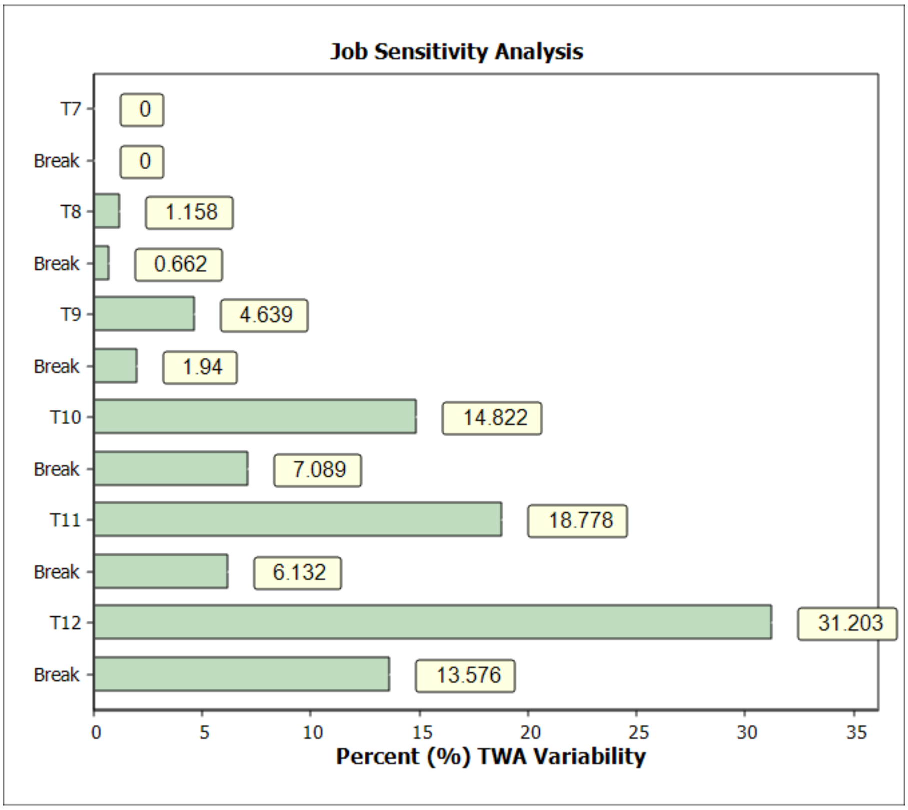
Job Sensitivity Analysis
T7
Break
T8
Break
T9
Break
T10
Break
T11
Break
T12
Break
0
0
1.158
0.662
4.639
1.94
14.822
7.089
18.778
6.132
31.203
13.576
0
5
10
15
20
25
30
35
Percent (%) TWA Variability

TEXT S7: Ag-HEC Near-Field RWC

Version: TEAS 2019 (Version 1.0)
Date: 11/25/2021 8:54:22 AM

SUBSTANCE INFORMATION

Ag-HEC particles dispersed in water. Ag content of aerosol particles at NF was 1.1% according to the ICP-MS analysis of collected particles. The fraction of Ag in the NF was used to calculate emission rate of Ag.

EXPOSURE LIMITS

Limit value	Type	Source	Notes
REL: 0.9 ug/m3	TWA	NIOSH REL	

TASK INFORMATION

RWC conditions: Room volume 600 to 735 m^3; General ventilation 1.25 to 5 ACH; NF volume min 0.5 m^3, mode 1.5 m^3, max 4 m^3; β = 2.8 m^3/min (GSD = 1.477).

TASK 1 - T1.1
Description: 1 nozzle
Model: M201
Model Name: 2Box.CE.Gv

Variable	Variable Model	Units
1: Frequency	~ ConstantInt (1, ,)	/shift
2: Duration	~ ConstantInt (180, ,)	min
3: G (Ag)	~ Uniform (0.0286, 0.0572,)	mg/min
4: Q	~ Uniform (1.25, 5,)	ACH
5: V	~ Uniform (600, 735,)	m^3
6: Vn	~ Triangular (1.5, 0.5, 4)	m^3
7: ß	~ Lognormal (2.8, 1.477,)	m^3/min

8: tg	~ Constant (180, ,)	min
9: t1	~ Constant (0, ,)	min
10: t2	~ Constant (180, ,)	min

TASK 2 - Break
Description: 1 nozzle
Model: M201
Model Name: 2Box.CE.Gv

Variable	Variable Model	Units
1: Frequency	~ ConstantInt (1, ,)	/shift
2: Duration	~ ConstantInt (60, ,)	min
3: G	~ Constant (0, ,)	mg/min
4: Q	~ Uniform (1.25, 5,)	ACH
5: V	~ Uniform (600, 735,)	m^3
6: Vn	~ Triangular (1.5, 0.5, 4)	m^3
7: ß	~ Lognormal (2.8, 1.477,)	m^3/min
8: tg	~ Constant (0, ,)	min
9: t1	~ Constant (0, ,)	min
10: t2	~ Constant (60, ,)	min

TASK 3 - T1.2
Description: 1 nozzle
Model: M201
Model Name: 2Box.CE.Gv

Variable	Variable Model	Units
1: Frequency	~ ConstantInt (1, ,)	/shift
2: Duration	~ ConstantInt (180, ,)	min
3: G (Ag)	~ Uniform (0.0286, 0.0572,)	mg/min
4: Q	~ Uniform (1.25, 5,)	ACH
5: V	~ Uniform (600, 735,)	m^3
6: Vn	~ Triangular (1.5, 0.5, 4)	m^3
7: ß	~ Lognormal (2.8, 1.477,)	m^3/min
8: tg	~ Constant (180, ,)	min
9: t1	~ Constant (0, ,)	min
10: t2	~ Constant (180, ,)	min

TASK 4 - Break
Description: 1 nozzle
Model: M201
Model Name: 2Box.CE.Gv

Variable	Variable Model	Units
1: Frequency	~ ConstantInt (1, ,)	/shift
2: Duration	~ ConstantInt (60, ,)	min

3: G	~ Constant (0, ,)	mg/min
4: Q	~ Uniform (1.25, 5,)	ACH
5: V	~ Uniform (600, 735,)	m^3
6: Vn	~ Triangular (1.5, 0.5, 4)	m^3
7: ß	~ Lognormal (2.8, 1.477,)	m^3/min
8: tg	~ Constant (0, ,)	min
9: t1	~ Constant (0, ,)	min
10: t2	~ Constant (60, ,)	min

TWA CALCULATION (TASK OR FULL SHIFT)

Task TWA - the TWA is calculated for the duration of the task(s).

SIMULATION OPTIONS

Unless noted below, for all variables modeled with a statistical distribution a random value was generated for each repeat of a task.

Room Variables:
- For each TWA, the room ventilation rate Q generated for the first Box model task was used for all of the following Box model tasks.
- For each TWA, the room volume V generated for the first Box model task was used for all of the following Box model tasks.

Options for 2Box variables t1, t2, and tg:
- If a random t1, t2 or tg > 0, apply following rules:
  1. if tg > Duration, let tg = Duration
  2. if t2 > Duration, let t2 = Duration
  3. if t1 > t2, let t1=1/2 t2

Options for 2Box Constant Emission Model variables t2 and tg:
- tg is not linked to task Duration (DEFAULT)
- t2 is always equal to task Duration (DEFAULT)

STATISTICS

Output Units:	μg/m3
Simulation N:	10000
Non-parametric Statistics	
Min:	4.456
Max:	40.410
Median:	12.765
Mode:	12.589
Fraction>REL:	100.000%
X0.05:	7.912
X0.50:	12.762
X0.60:	13.761
X0.70:	14.877
X0.75:	15.548
X0.80:	16.312
X0.90:	18.501
X0.95:	20.529
X0.99:	25.515
X0.999:	32.235
Normal Distribution Statistics	
Mean:	13.348
SD:	3.979
Lognormal Distribution Statistics	
GM:	12.797
GSD:	1.337
X0.95:	20.624
X0.99:	25.129
Fraction>REL:	100.000%

GRAPHS

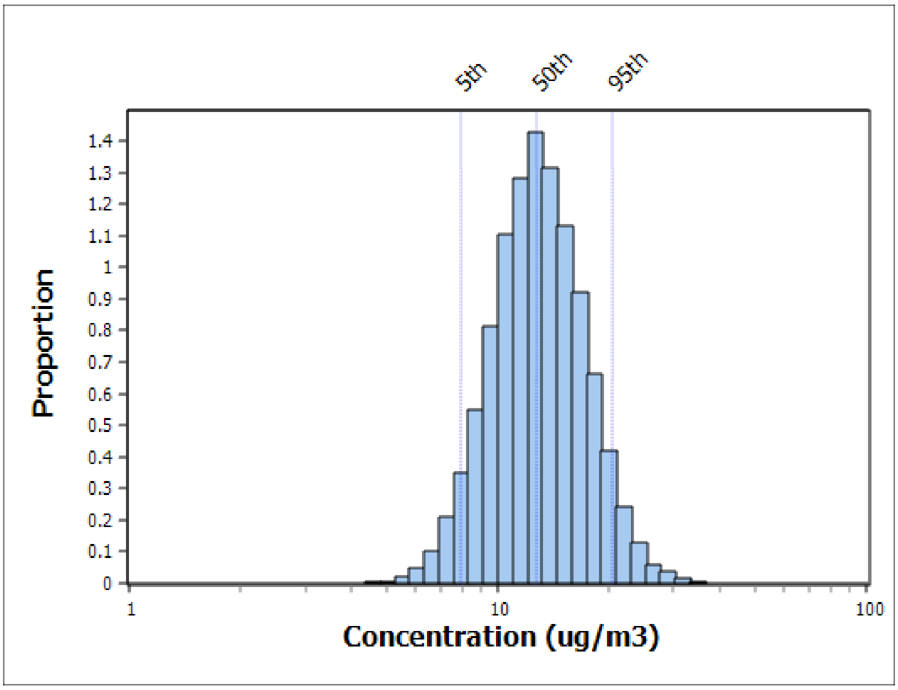
5th
50th
95th
Proportion
1.4
1.3
1.2
1.1
1
0.9
0.8
0.7
0.6
0.5
0.4
0.3
0.2
0.1
0
1
10
100
Concentration (ug/m3)

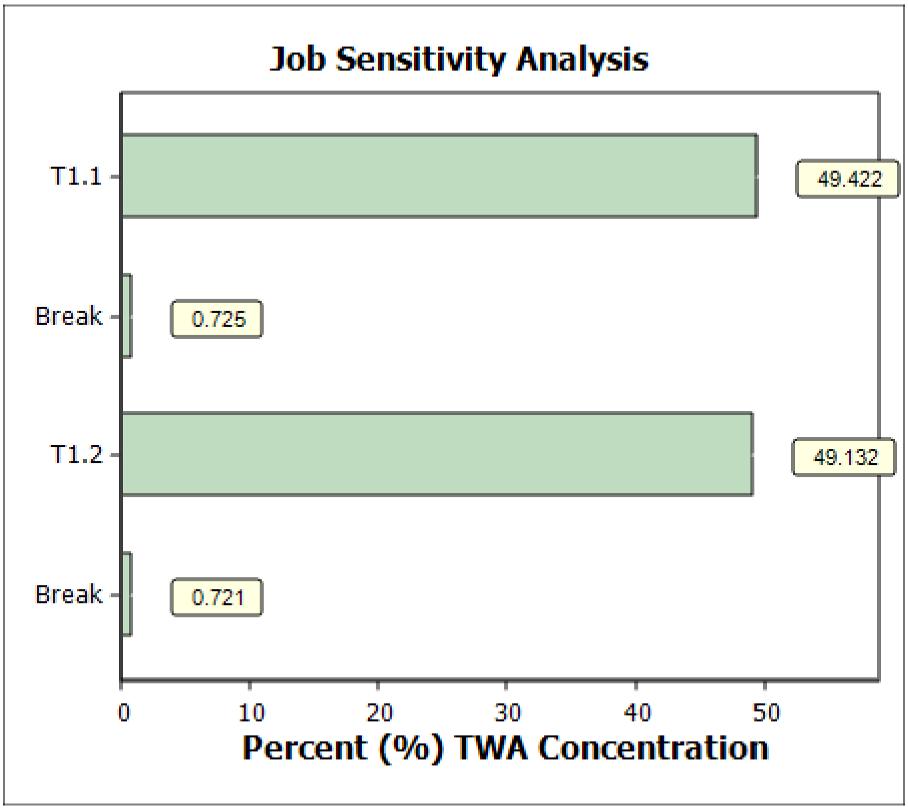
Job Sensitivity Analysis
T1.1
49.422
Break
0.725
T1.2
49.132
Break
0.721
0
10
20
30
40
50
Percent (%) TWA Concentration

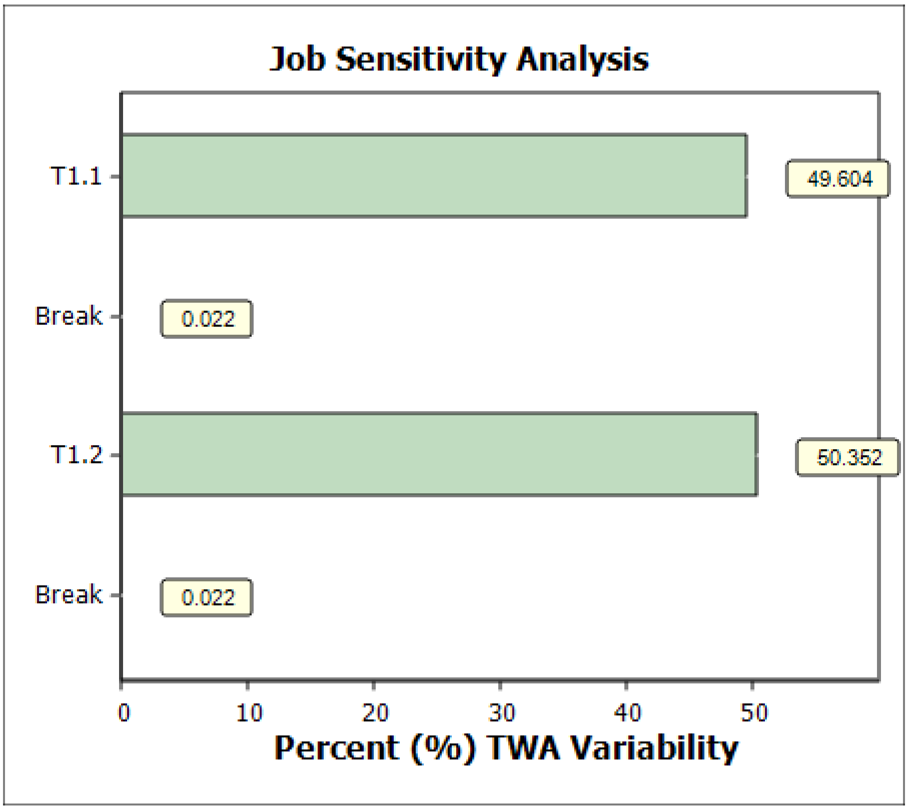
Job Sensitivity Analysis
T1.1
49.604
Break
0.022
T1.2
50.352
Break
0.022
0
10
20
30
40
50
Percent (%) TWA Variability
