# Supplementary material for: Quantifying Emission Factors and Setting Conditions of Use According to ECHA Chapter R.14 for a Spray Process Designed for Nanocoatings—A Case Study
Source: Nanomaterials (Basel). 2022 Feb 10;12(4):596. doi: 10.3390/nano12040596 (PMC8876979; doi:10.3390/nano12040596)
Supplement: Supplementary file 1 [file nanomaterials-12-00596-s001.zip › nanomaterials-1531087 - supplementary 3-proof done.pdf]

nanomaterials

Supplementary Material 3

Quantifying Emission Factors and Setting Conditions of Use According to ECHA Chapter R.14 for a Spray Process Designed for Nanocoatings—A Case Study

Optical. and mobility size of freshly generated TiO_2 agglomerates

Antti Joonas Koivisto [1,2,3,*], Benedetta Del Secco [4], Sara Trabucco [4], Alessia Nicosia [4], Fabrizio Ravegnani [4], Marko Altin [5], Joan Cabellos [6], Irini Furxhi [7,8], Magda Blosi [9], Anna Costa [9], Jesús Lopez de Ipiña [10] and Franco Belosi [4]

1 Air Pollution Management APM, Mattilanmäki 38, 33610 Tampere, Finland
2 Institute for Atmospheric and Earth System Research (INAR), University of Helsinki, PL 64, FI-00014 Helsinki, Finland
3 ARCHE Consulting, Liefkensstraat 35D, B-9032 Wondelgem, Belgium
4 CNR-ISAC, Institute of Atmospheric Sciences and Climate, National Research Council of Italy, Via Gobetti, 101, 40129 Bologna, Italy; b.delsecco@isac.cnr.it (B.D.S.); s.trabucco@isac.cnr.it (S.T.); a.nicosia@isac.cnr.it (A.N.); f.ravegnani@isac.cnr.it (F.R.); f.belosi@isac.cnr.it (F.B.)
5 Witek srl, Via Siena 47, 50142 Firenze, Italy; marko.altin@witekgroup.com
6 Leitat Technological Center, c/de la Innovació 2, Terrassa, 08225 Barcelona, Spain; jcabellos@leitat.org
7 Transgero Limited, Cullinagh, Newcastle West, Co. Limerick, V42 V384 Limerick, Ireland; irini.furxhi@transgero.eu
8 Department of Accounting and Finance, Kemmy Business School, University of Limerick, V94 T9PX Limerick, Ireland
9 ISTEC-CNR, Institute of Science and Technology for Ceramics, CNR, National Research Council, Via Granarolo 64, 48018 Faenza, Italy; magda.blosi@istec.cnr.it (M.B), anna.costa@istec.cnr.it (A.C.)
10 Basque Research and Technology Alliance (BRTA), Consiglio Nazionale delle Ricerche, Parque Tecnológico de Alava, Leonardo Da Vinci 11, 01510 Miñano, Spain; jesus.lopezdeipina@tecnalia.com (J.L.d.I.)
* Correspondence: joonas.apm@gmail.com; Tel: +358-407-222-029

Workplace exposure to TiO_2 nanoparticles is commonly measured by using differential mobility analyser (DMA) and optical particle sizer. Different metrics are usually combined without taking into account instruments different responses. Here is demonstrated with TiO_2 agglomerates the size relation between these two detection techniques. Freshly generated TiO_2 particles were size classified with DMA followed by optical sizing. Optical diameter was 0.14 times the mobility diameter for particles above 90 nm in diameter (detection limit of the optical particle sizer).

1. Introduction

Sizing accuracy of the OPC depends strongly on refractive index (*m*) of particle composition and particle morphology (Pinnick et al., 2000; Sorensen, 2001). Thus, the measurement results depend strongly on the aerosol properties. This may be eliminated with proper calibration to obtain comparable measurements between different detection techniques. Leskinen et al. (2012) performed a nanoparticle monitoring instrument comparison study, where additional experiment was performed by mobility classifying TiO_2 agglomerates and subsequently measuring with high resolution optical detector. This was used to assess the relation of TiO_2 agglomerates mobility and optical size.

2. Methods

TiO_2 agglomerates were synthesized in a laminar flow reactor by decomposing by a titanium tetraisopropoxide (97% TTIP solution, Aldrich) precursor (Koivisto et al., 2011; Leskinen et al., 2012). The experimental setup is presented in Figure S1.

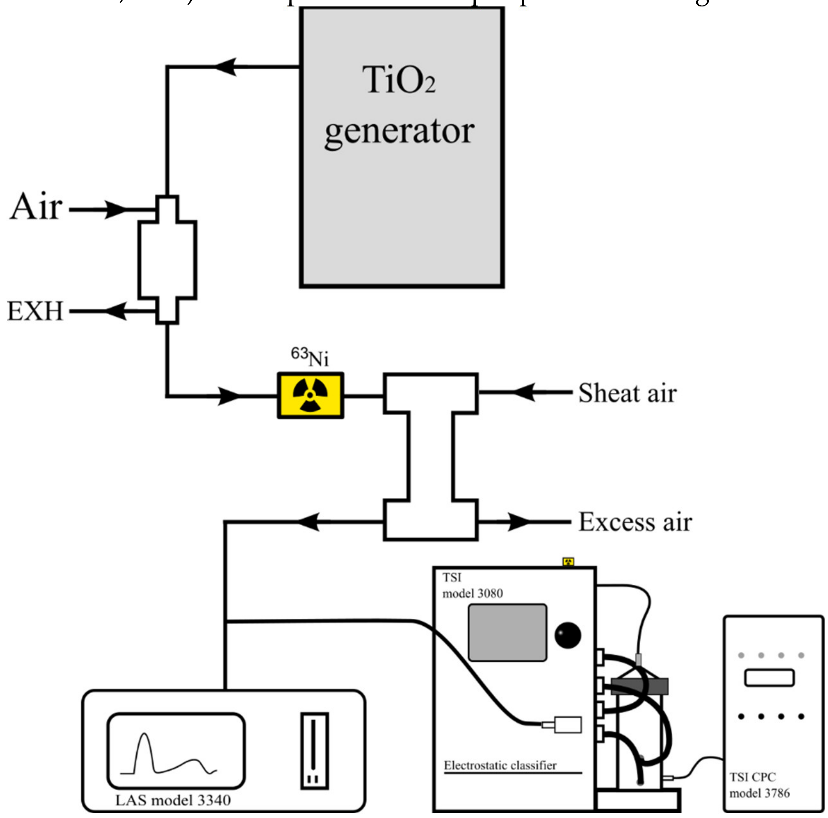

Figure S1. Experimental setup consisting of TiO_2 generator, a porous an ejector diluter (Lyyränen et al., 2004), ^{64}Ni neutralizer, a mobility classifier, and a scanning mobility particle sizer and laser aerosol spectrometer.

Particle optical diameter was measured with TSI Laser Aerosol Spectrometer (LAS) model 3340. The LAS uses a HeNe (λ = 633 nm) laser to illuminate particles with over 1 W power. Scattered light is collected with two pairs of Magnin collection optics (35-120°); one for avalanche photo diode to detect the smallest particles and another for PIN photodiode to detect large particles. This detection system ensures wide measurement range from 90 nm to 7.5 μm with high resolution (maximum 100 bins).

TiO_2 aerosol agglomerates with gas-phase nanoparticle generator (Leskinen et al., 2012). TiO_2 aerosol was neutralized with a bipolar ^{63}Ni charger and classified with Hauke short DMA. Aerosol classification was verified with TSI SMPS consisting of 3085 classifier with nDMA and 3786 CPC in parallel with LAS. The instruments factory calibrations were checked with polystyrene latex (PSL) particles (Leskinen et al., 2012). TiO_2 agglomerates mobility classification with DMA was verified the SMPS.

3. Results

The mobility classified TiO_2 particles correlation with the SMPS was

$$D_{TSI,mob} = -0.3 + 1.05 * D_{mob,classified}$$

This shows that the mobility classification was functioning properly. TiO_2 agglomerates optical size relation was

$$D_{opt} = 90\ nm + 0.14 \times D_{mob},$$

where the D_{opt} is the optical diameter (nm) and D_{mob} (nm) is the mobility classified TiO_2 agglomerate diameter. The individual measurements are shown in Figure 2.

4. Discussion and conclusions

Variation in Figure S2 may have been caused by mie-scattering non-linear behaviour (Pinnick et al., 2000). The relation of optical and mobility particle size can be used to translate mobility size to optical size or vice versa. The relation is useful when translating the particle size distribution to different metrics, such as surface area or mass (Koivisto, 2013). However, the relation was classified to highly agglomerated TiO_2 particles, which why the relation is not applicable to *e.g.*, spherical TiO_2 particles.

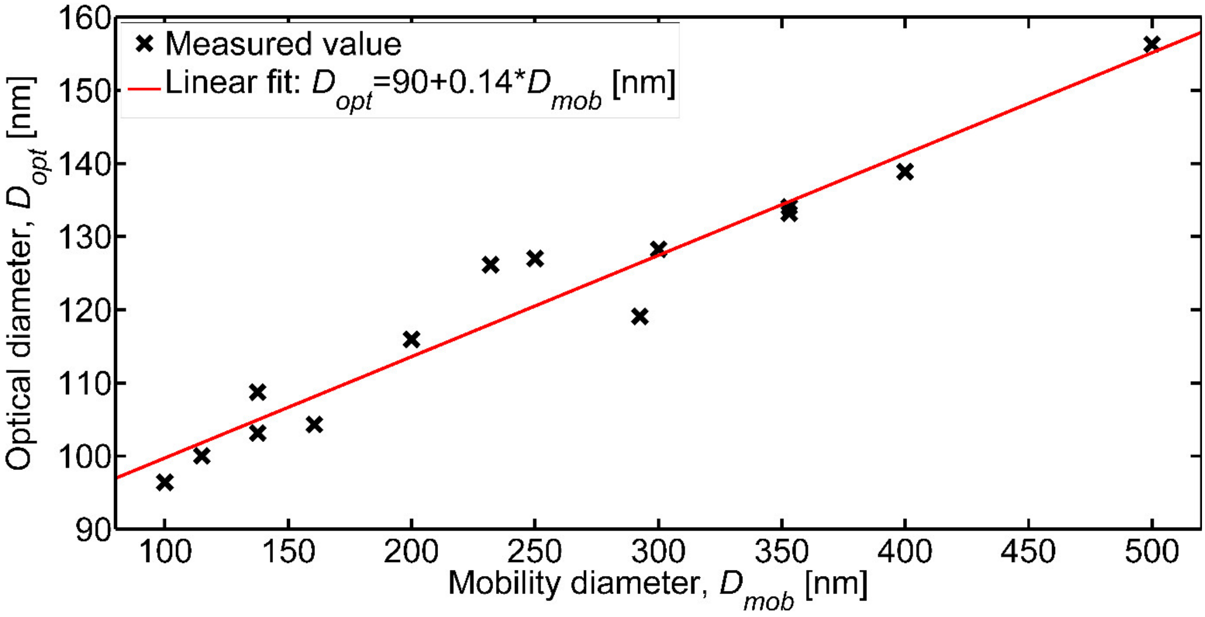

Figure S2. Optical diameter of mobility classified TiO_2 agglomerates. The refractive index of TiO_2 is $m_{TiO2} \approx 2.4$ at $\lambda = 630$ nm. The measured particle size distributions were narrow having a mean geometric standard deviation of 1.16.

Funding: This research was funded by the European Union's Horizon 2020 research and innovation programme under grant agreement No 862444.

Referecnes

Koivisto, A.J., 2013. Source specific exposure and risk assessment of indoor aerosols (No. 140), Report Series in Aerosolc Science. University of Helsinki, Helsinki, Finland.

Koivisto, A.J., Mäkinen, M., Rossi, E.M., Lindberg, H.K., Miettinen, M., Falck, G.C.-M., Norppa, H., Alenius, H., Korpi, A., Riikonen, J., Vanhala, E., Vippola, M., Pasanen, P., Lehto, V.-P., Savolainen, K., Jokiniemi, J., Hämeri, K., 2011. Aerosol characterization and lung deposition of synthesized TiO2 nanoparticles for murine inhalation studies. J Nanopart Res 13, 2949–2961. https://doi.org/10.1007/s11051-010-0186-x

Leskinen, J., Joutsensaari, J., Lyyränen, J., Koivisto, J., Ruusunen, J., Järvelä, M., Tuomi, T., Hämeri, K., Auvinen, A., Jokiniemi, J., 2012. Comparison of nanoparticle measurement instruments for occupational health applications. J Nanopart Res 14, 718. https://doi.org/10.1007/s11051-012-0718-7

Lyyränen, J., Jokiniemi, J., Kauppinen, E.I., Backman, U., Vesala, H., 2004. Comparison of Different Dilution Methods for Measuring Diesel Particle Emissions. Aerosol Science and Technology 38, 12–23. https://doi.org/10.1080/02786820490247579

Pinnick, R.G., Pendleton, J.D., Videen, G., 2000. Response Characteristics of the Particle Measuring Systems Active Scattering Aerosol Spectrometer Probes. Aerosol Science and Technology 33, 334–352. https://doi.org/10.1080/02786820050121530

Sorensen, C.M., 2001. Light Scattering by Fractal Aggregates: A Review. Aerosol Science and Technology 35, 648–687. https://doi.org/10.1080/02786820117868
